# Regulation of IFNβ expression: focusing on the role of its promoter and transcription regulators

**DOI:** 10.3389/fmicb.2023.1158777

**Published:** 2023-06-15

**Authors:** Jiqiang Fan, Qiumei Li, Jiadi Liang, Zhirong Chen, Linqin Chen, Junzhong Lai, Qi Chen

**Affiliations:** ^1^Fujian Key Laboratory of Innate Immune Biology, Biomedical Research Center of South China, Fujian Normal University, Fuzhou, China; ^2^The Cancer Center, Union Hospital, Fujian Medical University, Fuzhou, China

**Keywords:** IFNβ, regulatory element, promoter, enhancer, nucleosome, mechanism

## Abstract

IFNβ is a single-copy gene without an intron. Under normal circumstances, it shows low or no expression in cells. It is upregulated only when the body needs it or is stimulated. Stimuli bind to the pattern recognition receptors (PRRs) and pass via various signaling pathways to several basic transcriptional regulators, such as IRFs, NF-кB, and AP-1. Subsequently, the transcriptional regulators enter the nucleus and bind to regulatory elements of the IFNβ promoter. After various modifications, the position of the nucleosome is altered and the complex is assembled to activate the IFNβ expression. However, IFNβ regulation involves a complex network. For the study of immunity and diseases, it is important to understand how transcription factors bind to regulatory elements through specific forms, which elements in cells are involved in regulation, what regulation occurs during the assembly of enhancers and transcription complexes, and the possible regulatory mechanisms after transcription. Thus, this review focuses on the various regulatory mechanisms and elements involved in the activation of IFNβ expression. In addition, we discuss the impact of this regulation in biology.

## 1. Introduction

In 1957, Scientist discovered that a protein produced by embryonic chick cells could inhibit the replication of the live influenza virus. Since this protein interfered with viral infection it was named interferon. Currently, there are three types of interferons (IFN), namely, IFN-I, IFN-II, and IFN-III, with specific sub-categories (Meyts and Casanova, 2021; Sugrue et al., 2021). IFN-I is aggregated on human chromosome 9 and mouse chromosome 4 and includes IFNα, IFNβ, IFNε, IFNк, and IFNω (Bandurska et al., 2014; Gallucci et al., 2021). In this review, we will focus on IFNβ, which is a single-copy gene with a single and conserved sequence without an intron. It is normally suppressed or is present at very low levels in the body. In addition, IFNβ is a highly conserved key player in innate and adaptive antiviral immune responses (Chen et al., 2021; Zhang Y. et al., 2021), and is closely associated with the occurrence of various immune diseases and tumors (Sanford and McEwan, 2022; Yu R. et al., 2022). Additionally, IFNβ plays a vital role in growth and development, inflammation, immunity, diseases, and cancer.

IFNβ activation is necessary for cells to cope with environmental changes and is achieved via two types of activation mechanisms. One mechanism involves cytokines that play a role with specific modifications in the process of IFNβ regulation. The other mechanism depends on signaling pathways and has relatively fixed structural elements in the IFNβ promoter region. This mechanism has been extensively studied and various signal transduction pathways have been found. These pathways transmit immune signals to several types of transcription factors, such as IRFs, NF-кB, and AP-1, which are essential for regulating IFNβ expression. After a series of processing, the transcription factors translocate into the nucleus, bind to regulatory elements of IFNβ (positive regulatory domains I-IV, PRD I-IV), and finally mediate its expression and translation.

IFNβ is mainly regulated in its promoter region. Presently, structural elements that maintain low expression or suppress IFNβ in resting cells and various conservative structural elements that activate transcription have been found. Moreover, new distal enhancer elements, which are necessary for IFNβ expression have been found. Under the joint action of various regulatory elements and factors, several stages of reinforcement assembly, chromatin remodeling, nucleosome sliding, and transcriptional holoenzyme complex formation are completed to activate IFNβ expression. In addition, a new regulatory mechanism of IFNβ before the beginning of post-transcriptional translation has been found. In this paper, we review the structural information and various mechanisms involved in the expression regulation of IFNβ in detail. Additionally, we summarize the newly discovered regulatory elements and mechanisms of IFNβ.

## 2. IFNβ transcription regulation

### 2.1. Basic structure of IFNβ promoter

**Figure 1:**
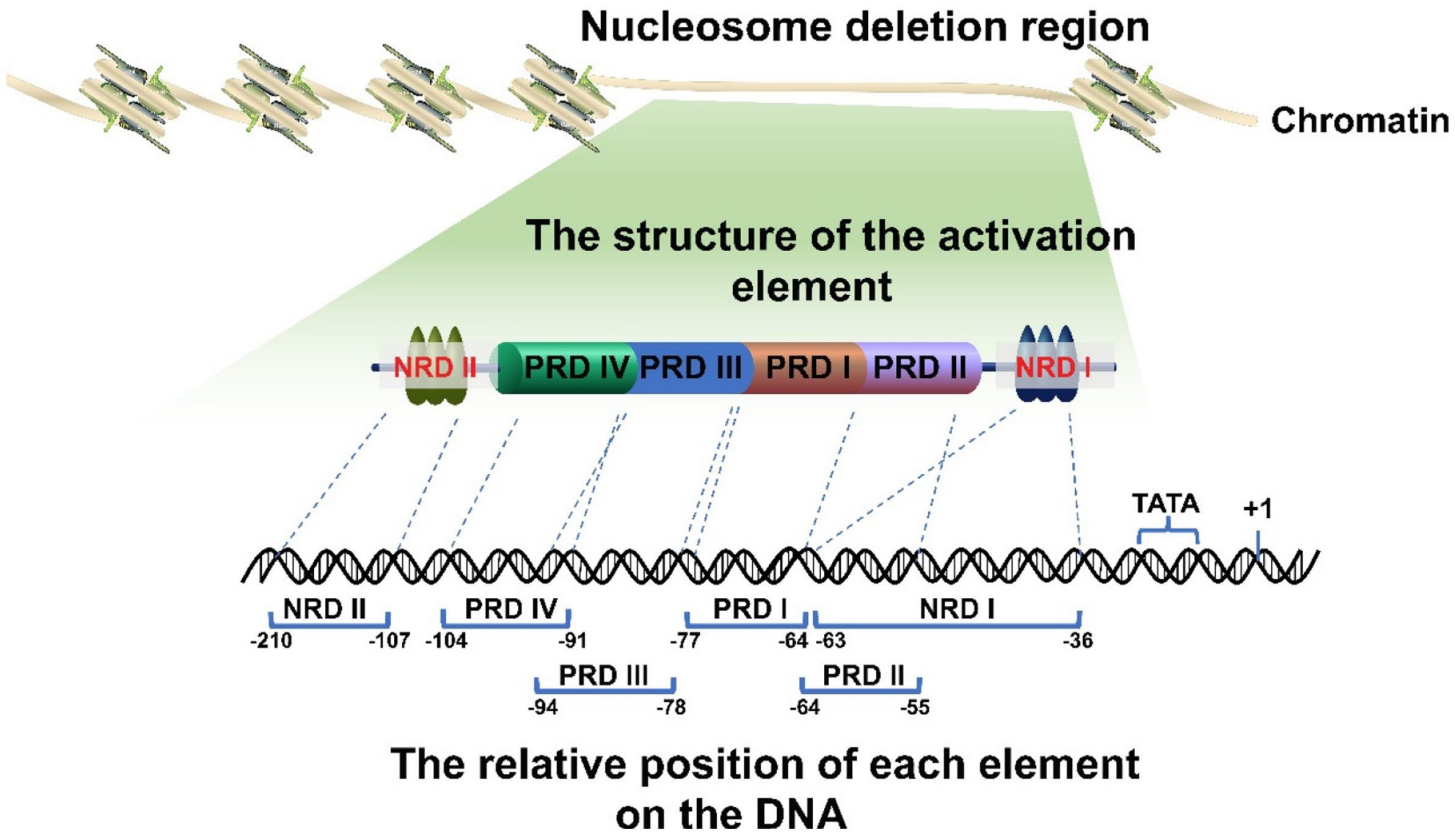
Schematic diagram of the IFNβ promoter. The promoter is located in the deletion region of the nucleosome. It has multiple regulatory elements with clearly determined positions. Their correct arrangement is crucial for regulation. In addition, the promoter region includes a nucleosome II, which masks the TATA box and transcription initiation site, thereby leaving the promoter in a repressed state.

There are various proximal and distal regulatory sites in the IFNβ promoter. In addition, the IFNβ promoter is located in a nucleosome deletion region which provides transcription factor binding sites and facilitates recognition by transcription initiation enzymes to activate gene expression. Thus, different genes will be specifically activated by different transcription factor binding sites within this region (Fragoso and Hager, 1997; Agalioti et al., 2000; Lomvardas and Thanos, 2001; Lee et al., 2007). The proximal regulatory elements of the IFNβ gene (called the basal transcriptional regulatory regions) have been accurately located (Figure 1). They mainly consist of four positive regulatory domains (PRD I-IV), a nucleosome (nucleosome II) covering the TATA box to prevent transcription recognition, and two negative regulatory domains (NRDI and NRDII). Of these, PRD I (−77 to −64) (Fujita et al., 1988; Goodbourn and Maniatis, 1988; Leblanc et al., 1990; Doly et al., 1998) and III (−94 to −78) (Leblanc et al., 1990; Panne et al., 2004) interact with numerous interferon regulatory factors (IRFs) and CREB binding protein CBP/p300 to form a complex. PRD II (−64 to −55) (Goodbourn and Maniatis, 1988; Fan and Maniatis, 1989; Leblanc et al., 1990) binds to NF-кB and PRD IV (−104 to −91, the key sequence is −99 to −91, partially overlapped with PRD III) (Fujita et al., 1985; Du and Maniatis, 1992) binds to AP-1. The repressor protein binds to NRDI (approximately at −63 to −36, with the 5′ end partially overlapping the PRD II and the boundary is uncertain) (Goodbourn et al., 1986; Goodbourn and Maniatis, 1988; Fujita et al., 1989) and NRDII (−210 to −107) (Zinn et al., 1983; Whittemore and Maniatis, 1990a,b). This represses the gene and stimulates signals that inactivate or translocate the repressor binding to NRDI and NRDII, thus allowing transcription to occur (Whittemore and Maniatis, 1990a). The distal regulatory elements of IFNβ are relatively newly discovered enhancers, which also play a key role in IFNβ expression.

### 2.2. Transcription factors involved in IFNβ expression

**Figure 2:**
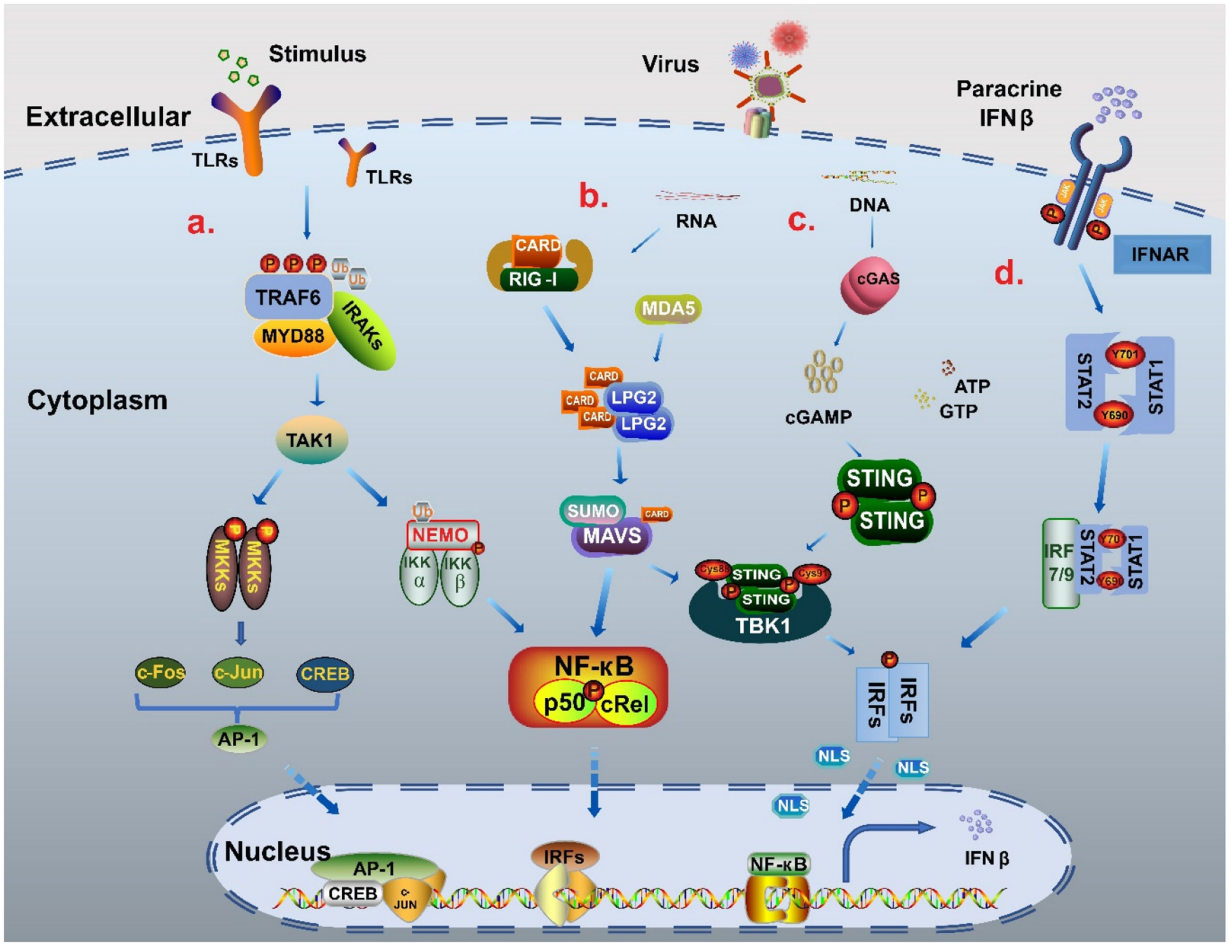
The signal pathway regulatory network of IFNβ. **(A)** NF-κB pathway and MAPK pathway When stimulated, TLRs recruited myeloid differentiation primary reactive protein 88 (MyD88) and IL-1 receptor-related kinases (IRAKs), and then bound tumor necrosis factor (TNF) receptor-related factor 6 (TRAF6) to promote phosphorylation and polyubiquitination to activate TAK1. On the one hand, through ubiquitination, TAK1 binds to the catalytic subunits IKKα, IKKβ and regulatory subunits NEMO of the IKK complex, and finally forms two NF-кB heterodimers p50: RelA and p50: cRel into the nucleus to participate in gene expression. TAK1 activates MAPK kinase family members (MKKs) to produce c-Jun, c-Fos and CREB through AMPK pathway, and then forms polymeric complexes that bind to the promoter region to regulate the expression of IFNβ (Farooq et al., 2021; Duan et al., 2022). **(B)** MAVS-RIG pathway When the stimulus signal is detected, the conformation of RNA sensor RIG-I and MDA5 change to release the signal domain CARD. MAVS form MAVS complex with CARD in coordination with LPG2, which can cause the cascade reaction of TBK1/IRFs and also increases the activation of NF-кB to promote IFNβ expression (Thoresen et al., 2021; Xu S. et al., 2022). **(C)** cGAS-STING pathway When recognizing DNA, cGAS catalyzes the reaction of adenosine triphosphate (ATP) and guanosine triphosphate (GTP) to form GMP-AMP (cGAMP). cGAMP binds to STING as a second messenger, causing STING to be palmitoacylated at two cysteine residues (Cys88 and Cys91), while recruiting TBK1 to form a complex. This complex triggers the phosphorylation and dimerization of IRF3, which then enters the nucleus to promote IFNβ production (Zhang K. et al., 2021; Deater et al., 2022). **(D)** JAK–STAT pathway The combination of parathine IFNβ and receptor IFNΑR leads to recruitment of STAT1 and STAT2 by JAK, which phosphorylates Tyr701 of STAT1 and Tyr690 of STAT2 to form heterodimers that interact with IRF9 to form the trimer complex ISGF3. ISGF3 can increase IFNβ expression through transcription factors such as IRF3/7 in a positive feedback way (Michalska et al., 2018; Gao T. et al., 2021).

Cells respond to immune-related external stimuli and intracellular substances through a series of signaling pathways. Pattern recognition receptors (PRRs) bind to pathogen-associated molecular patterns (PAMPs) or host-derived damage-associated molecular patterns (DAMPs) (Webb and Fernandez-Sesma, 2022), which is the first step in signal transduction. The PRRs include Toll-like receptors (TLRs), Rig-like receptors, NOD-like receptors (NLRs), AIM2-like receptors (ALRs), C-type lectin receptors, and intracellular DNA and RNA sensors (Uehata and Takeuchi, 2020; Chen et al., 2021). Multiple pathways, such as MAPK, cGAS-STING, MAVS-RIG, and JAK–STAT, are involved in IFNβ regulation and expression. Based on the stimulator binding the receptor, signals are transmitted via different signaling pathways to several basic transcriptional regulators, including the interferon regulatory factor (IRF) family, NF-кB family, and AP-1 family, that regulate IFNβ expression. Subsequently, the transcription factors work together with the promoter regulatory elements to regulate IFNβ expression (Figure 2). Therefore, we focus on these transcription factors and deeply analyze their structures and processes by which they regulate IFNβ.

#### 2.2.1. IRF family

There are nine species of IRFs in mammals and a specific IRF10 is found in birds and bony fish (Suzuki et al., 2011; Negishi et al., 2018), which have significant homology. Their N-terminal region (∼120 amino acids long) is a DNA binding domain (DBD) (Antonczyk et al., 2019; Jefferies, 2019) and has five repeat sequences composed of tryptophan, of which three are connected to the DNA sequence called the interferon-sensitive response element (ISREs) (Cook et al., 2020). The C-terminal contains an IRF-association domain (IAD), which consists of a nuclear export sequence, an autoinhibitory sequence, and an IRF binding domain (Jefferies, 2019). The IRF family members homodimerize or heterodimerize to exert transcriptional or inhibitory activity.

Currently, all IRF molecules except IRF4/6 are thought to be directly or indirectly involved in the regulation of IFNβ expression (Jefferies, 2019).

IRF3 is one of the most important transcription factors directly participating in the regulation of the IFNβ promoter. It is a 47 kDa protein with a length of 427 amino acids (AA). The ISRE site of IRF3 interacts with the promoter sequence 5′-GAAANNGAANN-3′ of IFNβ (N = A, C, G, or T) (Ysebrant de Lendonck et al., 2014). The presence of a serine-rich auto-inhibition element (AIE) (Sancho et al., 2009) at the C-terminal makes IRF3 inactive under static conditions. In addition, the DBD region of IRF3 contains a nuclear localization signal (NLS) and a nuclear exit signal (NES) that shuttle IRF3 between the nucleus and cytoplasm. Since NES acts dominantly, IRF3 mainly exists in the cytoplasm in the steady state (Ysebrant de Lendonck et al., 2014). When stimulated, IRF3 is rapidly activated and translocated to the nucleus, where it binds to the IFNβ promoter to mediate its expression. Additionally, IRF3 activation requires the cooperation of cofactors. On the one hand, Bromodomain protein 3 (Brd3) interacts with IRF3 and p300 to increase the acetylation of IRF3 mediated by p300. On the other hand, it can promote the recruitment of IRF3/p300 complex on IFNβ promoter (Ren et al., 2017). However, IRF3 activation is suppressed by NFAT5, a negative regulator, which can competitively bind to the key conservative sequence of IRFs in the IFNβ promoter, thus keeping IFNβ returned to a low level after stimulation (Huerga Encabo et al., 2020). In addition, KLF4 can also inhibit the recruitment of IRF3 to the IFNβ promoter during virus infection by promoting its transfer from the cytoplasm to the nucleus and binding to the IFNβ promoter (Luo et al., 2014). Similarly, the TAB1 protein can disrupt IRF3 binding by recruiting histone deacetylase 8 (HDAC8) to the IFNβ promoter as well (Yu Z. et al., 2022).

IRF7 is the closest family member of IRF3. The recruitment of the MyD88 and IRAK1/2/4 signaling complex leads to IKKα activation and IRF7 phosphorylation, which is involved in IFNβ regulation mediated by IRF3. However, only a small amount of IRF7 is present in the early stage of IFNβ induction, at which IRF3 plays a major role. IRF3 is degraded in the late stage of IFNβ induction while a large amount of IRF7 is induced to play a major regulatory role (Ning et al., 2011). In addition, the activation of RIG-I/MAVS signal pathway induced by RNA virus can lead to IRF7 phosphorylation and promote IFNβ expression (Tang and Wang, 2009). For example, Wu et al. (2020) found that influenza A virus (IAV) activates RIG-I and IRF7, and the activation of IRF7 is necessary for IFNβ production in the second stage of viral infection. Ling et al. (2019) found that TAR RNA binding protein 2 (TARBP2) inhibits K63-linked ubiquitin between IRF7 and TRAF6, which is a prerequisite for IRF7 phosphorylation, resulting in inhibition of IFNβ expression. IRF1/2 is mainly expressed in lymphocytes, macrophages, granulocytes and NK cells. IRF1 is a positive regulator activated by IFNAR/JAK/STAT signal axis. IRF1 promotes IRF3 phosphorylation and participates in the regulation of IFNβ expression by blocking the binding of IRF3 and protein phosphatase 2A (PP2A). IRF2 can competitively bind with IRF1 to inhibit the expression of IFNβ in the immune process, and play an important role in promoting the development and maturation of some cells (Kawasaki et al., 2014; Du et al., 2022; Persyn et al., 2022; Wen et al., 2022). IRF5 was only expressed in monocytes, macrophages and dendritic cells. IRF5 is activated by RIG-I-like receptor signaling and phosphorylated by IKKβ at conserved residues in its IAD domain. This leads to its nuclear translocation where it interacts with transcriptional coactivators such as CBP/p300 in promoting interferon production (Jefferies, 2019). Besides, IRF5 is also a key member of IFNβ expression in TLR8 signal transduction pathway (Jefferies, 2019; Nilsen et al., 2022). IRF8 was initially discovered to play a role in the induction of pro-inflammatory genes. However, recent studies have shown that it is also involved in the induction of interferon in dendritic cells. It assists in the prolongation of the recruitment of the basic transcription mechanism of the promoter (Jefferies, 2019). IRF9 is the target of IFNβ. It interacts with heterodimers STAT1 and STAT2 to form a new trimer complex, ISGF3, which is involved in downstream gene regulation (Michalska et al., 2018; Platanitis and Decker, 2018; Yu R. et al., 2022). IRF10 shows obvious species specificity and is a negative regulator that inhibits the type I IFN promoter response mediated by RIG-I, MDA5, TBK1, and MITA (Chen et al., 2017).

#### 2.2.2. NF-кB family

NF-кB is a DNA-binding transcription factor family (Mulero et al., 2019a) that exists in the dimer form made up of five related polypeptide subunits, namely, RelA (p65), RelB, cRel, p50, and p52. They form DNA target-specific homodimers or heterodimers through various combinations and play numerous functions (Mulero et al., 2019b; Chawla et al., 2021). The most common NF-кB dimers involved in INFβ regulation are the RelA: RelA homodimer, p50: RelA heterodimer, p50: cRel heterodimer, cRel: cRel homodimer, p50: RelB heterodimer, and p52: RelB heterodimer (Mulero et al., 2019b). Although not all NF-кB dimers have been implicated in the regulation of IFNβ, existing studies suggest that different dimers can be produced through different signaling pathways. The classical NF-кB pathway primarily generates two NF-кB heterodimers, p50: RelA and p50: cRel (Sun, 2017). On the other hand, the NEMO independent non-classical pathway relies on NF-κB-induced kinase (NIK, also known as MAP3K14) and IKKα to activate (Jin et al., 2014; Sun, 2017) the NIK/IKKα complex via signal transduction. This complex then phosphorylates and ubiquitinates p100, which is processed into p52. The resulting p52: RelB heterodimers are involved in IFNβ regulation (Sun, 2017; Haga and Okada, 2022).

Each NF-κB subunit has a highly conserved N-terminal region of approximately 300 residues called the REL homologous region (RHR) containing two independent structural elements, the N-terminal domain (NTD) and the NLS. The NLS allows entry into the nucleus, while the NTD binds to a 5′-GGGNNNNNCC-3′ specific sequence of 5–11 bps (Located in PRD II area) on the IFNβ double-stranded DNA (Haga and Okada, 2022). Therefore, this DNA sequence is known as the кB DNA/site. NF-кB can bind to several gene promoters or DNA sequences of invalid fragments. Hence, how does it specifically activate IFNβ expression is questionable? Nikopoulou et al. (2019) have a good explanation for this query. They revealed that exogenous or endogenous stimulation induces ThPOK, a transcription factor, to bind to NRC21 on multiple sites, including the conservative GAGA elements that facilitate interchromosomal interaction. Under the synergistic action of ThPOK, NRC21, an Alu-like DNA element (Apostolou and Thanos, 2008; Deininger, 2011), can capture NF-кB to form special GAGA-кB elements known as the NF-кB reception centers. NRC21 coincides with the NF-кB binding site in the IFNβ promoter and/or overlaps the ThPOK-binding site. Thus, free NRC21 can gather in the IFNβ promoter region and NF-кB binding to NRC21 can be transferred to the IFNβ PRD II binding site through interchromosomal interaction. In addition, they confirmed that ThPOK and NF-кB bind to NRC21 earlier than IFNβ enhancers. Moreover, DNA binding synergism between NF-кB and ThPOK requires complete кB and GAGA sites, which is essential for the initiation of IFNβ. Interestingly, IFNβ activation, regulated by NF-кB, maintained in a reasonable range can effectively avoid the harmful consequences of excessive inflammation. Keap1 can bind to IFNβ and induce G9a-GLP, NF-кB p50, and H3K9me2 recruitment to reduce inflammation. However, it does not affect the recruitment of NF-κB p65, IRF3, or c-Jun; hence, the effect of reducing inflammation is limited (Burns and Kerppola, 2022).

#### 2.2.3. AP-1 family

Activating protein-1 (AP-1) is a complex transcription factor existing in a dimer form. It consists of members of four protein families: Jun (c-Jun, Junb, and Jund), Fos (c-Fos, FosB, Fra-1, and Fra-2), ATF (ATF-2, ATF-3/LRF1, ATF-4, ATF-5, ATF6B, ATF-7, BATF, BATF-2, BATF-3, and JDP2), and MAF (c-MAF, MAF-A, MAF-B, MAF-F, MAF-G, MAF-K, and NRL) by homodimers and heterodimers (Bejjani et al., 2019; Bhosale et al., 2022). AP-1 participates in many processes, such as cell proliferation, differentiation, apoptosis, transformation, migration, and survival (Hess et al., 2004). In addition, it is involved in the regulation of IFNβ expression, mainly controlled by the MAPK and NF-кB pathways (Bhosale et al., 2022).

AP-1 mediates the expression of many genes and exhibits transcriptional universality. Further studies are needed to investigate which dimer assemblage is involved in regulating of IFNβ expression. Here, we summarize some studies that can specifically regulate the expression of IFNβ through a single pathway. For instance, the retinoblastoma protein (Rb) and c-Jun: ATF-2 heterodimer subgroup c-Jun interacts with the IFNβ enhancer region. HDAC1 and HDAC8 inhibit histone H3/H4 acetylation in the IFNβ promoter, thus inhibiting its transcription (Meng et al., 2016). In macrophages, ATF3 is a transcriptional suppressor and a key component of IFN negative feedback regulation. It binds competitively to a positive regulator on the distal enhancer, 15 kb upstream of the IFNβ promoter, and interacts with histone deacetylase 1 (HDAC1) to counteract the action of histone acetyltransferase, maintaining a closed chromatin conformation to restrict transcription (Labzin et al., 2015).

### 2.3. Several distal enhancers

Gene enhancers do not limit the vicinity of gene promoters but exist upstream, downstream, or distal to the gene. Through chromosome conformation capture (3C), chromatin status assessment, and sequence analysis, Banerjee et al. (2014) found L2 enhancer 19.7 kb upstream of IFNβ. It contains DNase I hypersensitive sites (DHS) that have been modified by H3K4me1 and H3K27ac, which are necessary to enhance the transcription activation of target genes. Additionally, an ISRE combination sequence was found here. With the help of the IRF3 binding motif, phosphorylated-IRF3 is recruited and cooperates with the IFNβ proximal promoter region and enhancers to induce its expression. Interestingly, L2 enhancer exhibits activities of virus-induced enhancers and bidirectional promoters. Additionally, it produces virus-induced eRNAs, which are bidirectional transcribed RNAs generated by enhancers with enhancer activity and bidirectional promoter activity. And, they also found that eRNAs have significant activity *in vivo*, and the production of their specific sequences is essential to maintain their activity and may be regulated by IRF3. Irrespective of the direction, two base mutations in the ISRE of IRF3 hinder the activity of the L2 promoter and enhancer. Moreover, studies showed that eRNAs can affect the chromosome ring at the molecular level, and a decrease in eRNA will reduce IFNβ expression. This indicates that the production of eRNAs is strongly correlated with high IFNβ expression. Subsequently, based on this, Ferri et al. (2015) found that ICE (An enhancer that may be the same as L2) has an open chromatin structure. Due to their similar action position and function, ICE and L2 should be considered as the same enhancer. However, its position is different from that defined by Banerjee et al., where ICE is located 15 kb upstream of IFNβ. ICE is highly conserved during myeloid differentiation, and contains PU.1 binding sites and DNA binding sites of transcriptional regulators, such as IRF3, c-Jun, or p65. PU.1 recruits TRIM33, a ubiquitin protein, to ICE by preventing CBP/p300 recruitment, thereby regulating the load of IFNβ enhancers, controlling the structure of IFNβ chromatin, and eventually shutting down IFNβ gene transcription. However, this regulation occurs only at the late stage of Toll-like receptor-mediated macrophage activation and does not affect initial IFNβ expression.

Strikingly, Assouvie et al. (2020) found that high IFNβ expression in mouse myeloid cells is related to increased cyclization of 100 kb (partially overlapping with Ptplad2/Hacd4) downstream region of IFNβ and ICE. This region was identified as the myeloid super enhancer and consists of three separate enhancers, one of which responds to IFNβ regulation and is named FIRE; however, this is uncertain in other cell lines. This super enhancer contains a lipopolysaccharide-induced enhancer and has several single nucleotide polymorphisms (SNPs). Its activity depends on the binding of CEBPB. Interestingly, the human homologous gene carries an IFNβ eQTL, a genetic polymorphism associated with differential IFNβ expression. rs12553564, an SNP reported in the FIRE region of IFNβ, could disrupt a conserved CEBPB binding motif leading to differential IFNβ expression.

Zeng et al. (2010) found, there is an XBP-1-dependent enhancer in the +6.1 kb conserved region downstream of IFNβ, which contains bonding sites for XBP-1, IRF3, and CBP/p300. Perturbations in the endoplasmic reticulum (ER) result in a conserved stress response called the “Unfolded Protein Response (UPR)” that can regulate XBP-1. In the presence of UPR and LPS stimulation, XBP-1 binds to the TGCA core motif on this enhancer and then binds to IRF3, CBP/p300 in this region to form a large complex. This complex forms a chromatin ring that folds to the proximal promoter of IFNβ, enhancing recruitment and thus increasing IFNβ expression. However, this process functions only when UPR effect exists.

Currently, there are limited studies on the regulation of distal enhancers of IFNβ, and no drugs have been found to interfere with this process. However, since the distal enhancer is far away from the traditional transcriptional regulatory region, it is not easy to interfere with other regulatory elements, but rather functions as an enhancement role. Therefore, developing drugs that target the distal enhancer sequence or regulatory process may be significant for treating some diseases that involves overexpression of IFNβ. For instance, abnormal activation of the cGAS-STING pathway causes overexpression of INFβ in autoimmune diseases.

### 2.4. Various forms of modification regulation

Studies have found that various modifications, such as phosphorylation, acetylation, ubiquitination, methylation, SUMOylation, and non-coding RNA regulation, regulate IFNβ expression (Chiang et al., 2021; Wang and Ning, 2021). These modifications can be roughly divided into three categories: regulatory region sequence modification, transcription factor binding modification, and mRNA direct modification. In this section, we summarize the latest progress witnessed.

Three types of methylation, namely, DNA methylation, mRNA methylation, and protein methylation, are directly involved in the regulation of IFNβ expression. DNA methylation refers to the addition of a methyl group to the C5 position of cytosine to form 5-methylcytosine (5mC) and is catalyzed by DNA methyltransferases, such as DNMT3a and DNMT3b (Chen et al., 2017; Tan et al., 2022). Gao Z.J. et al. (2021) found a CpG single nucleotide methylation 100 bp away from the IRF3 binding sequence. This methylation inhibits the recruitment of IRF3 to PRD III and PRD I by disrupting the binding of IFNβ-related transcription factors and its motif, resulting in the inhibition of IFNβ expression by blocking promoter region binding. Wang et al. (2023) found that DNA methylation regulator UHRF1 could negatively regulate the expression of IFNβ by regulating the phosphorylation of IRF3. Ptaschinski et al. (2015) found that demethylation enzyme KDM5B can also participate in the regulation of IFNβ expression, but the specific mode of action remains to be further studied. N6-methyladenosine (m6A) is mainly distributed in the mRNA coding sequence (Rubio et al., 2018; Tong et al., 2022). Rubio et al. (2018) found that the coding sequence and 3′ untranslated region (UTR) of IFNβ are modified by m6A, which is controlled by M6A methyltransferase subunit METTL14 and demethylase ALKBH5, thus directly act on the mRNA of IFNβ and participates in translation regulation and mediates the stability of mRNA. The deletion of METTL14 can promote the expression of IFNβ, while ALKBH5 deletion causes an opposite effect. Protein methylation primarily occurs in histones. Disruptor of telomeric silencing-1-like (DOT1L) is a unique H3K79 methyltransferase. When signal pathway is activated, the levels of H3K79me2/3Di/tri-methylation modifications on the IFNβ promoter significantly increase by DOT1L (Chen et al., 2020). In addition, the interaction between MLL4 and demethylase Kdm6a promotes the expression of IFNβ enhancers (Li et al., 2017). However, methylation in non-histone proteins, such as protein arginine methyltransferase 1 (PRMT1), has recently been found to participate in regulation (Chen et al., 2017).

Histone acetylation also play a critical role in regulating IFNβ expression. During viral infection, histone deacetylase 9 (HDAC9) deacetylates tank-bound kinase 1 (TBK1) to activate TBK1 phosphorylation, leading to an increase in type I IFN. IRF9 acetylation by CBP on K81, is necessary for IRF9 to bind to STAT1 and activates IFNβ expression (Chen et al., 2017). Recent studies, Liu et al. (2016) have demonstrated that ASF1a promotes IFNβ production of IFNβ by promoting the CBP-mediated acetylation of H3K56.

At present, microRNAs (miRNAs) and lncRNAs are main non-coding RNAs that regulate gene expression (Muntyanu et al., 2021; Chen et al., 2022; Ji et al., 2022). Lnc-MxA is a non-coding RNA that directly interacts with the IFNβ promoter, thereby forming an RNA–DNA triplex with the IFNβ promoter. This hinders the binding of IRF3 and NF-кB and effectively inhibits IFN I activation (Suarez et al., 2020). Additionally, lncRNA NKILA directly blocks IκB phosphorylation and interacts with NF-κB to form a stable ternary complex NF-κB/IκB/NKILA. The TNF-α-induced pseudogene Lethe binds to NF-κB p65/RelA subunits and blocks their binding to the IFNβ promoter, thus reducing inflammation (Suarez et al., 2020). Moreover, Malat1 selectively promotes the production of antiviral IFNβ by increasing the level of nuclear IRF3 protein (Liu et al., 2020). The small ubiquitin-like modifier (SUMO) encodes the proximal enhancer of the IFNβ gene and thus inhibits the basic activity of the IFNβ promoter (Decque et al., 2016).

## 3. Reinforcement assembly

**Figure 3:**
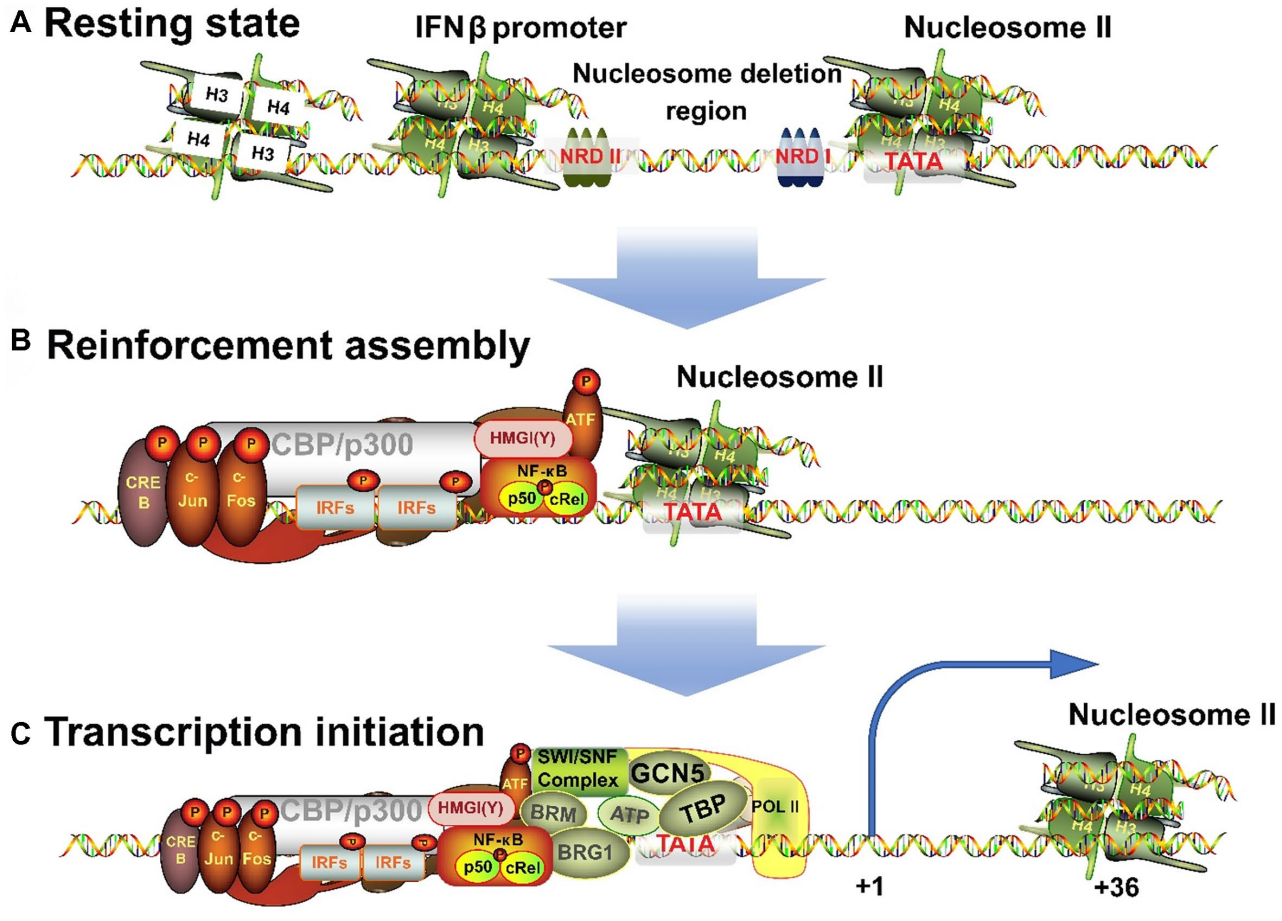
Three processes for the transition of IFNβ promoter from a resting state to an activated state. **(A)** When unstimulated, the cells are in a resting state, and NRDI and NRDII bind to the promoter region and suppress transcription. **(B)** After receiving external signals, the stimulus is transmitted through signaling pathways to transcription factors, which are transferred to the nucleus to combine with the corresponding elements to form an enhancer. **(C)** The completion of the assembly of enhancers will continue to recruit chromatin remodeling related enzymes, shift the nucleosome downstream to expose the TATA box and transcription starting point, and induce RNA polymerase II binding to form a holoenzyme complex and activate transcription.

The IFNβ transcription begins with the assembly of nucleosome deletion enhancers (Vinayachandran and Bhargava, 2022; Figure 3A), phosphorylation of IRFs to form dimers after their activation, and translocation of IRFs to the nucleus where they bind to PRD I and III of IFNβ promoter. The ISREs of IRFs bind to consensus binding sequence 5′-AANNGAAA-3′ (core motif 5′-GAAA-3′) on IFNβ and to CBP/p300, which can also bind c-Jun, ATF-2, and NF-кB (Fujita et al., 1987; Panne et al., 2004). However, miRNAs can inhibit CBP/p300. Fortunately, Qu et al. (2021) found a new intron circRNA, named AIVR, which acts as a miRNA sponge to remove the inhibition.

High mobility group (HMG) proteins are structural DNA and nucleosome binding proteins and can be subdivided into three families: HMGA (HMGI/Y/C), HMGB (HMG1/2), and HMGN (HMG14/17) (Hill et al., 1999; Pogna et al., 2010). HMGA is involved in the transcriptional regulation of IFNβ. HMG-I (Mr 11.7 kDa) and HMG-Y (Mr 10.5 kDa) are homologous proteins produced by alternative splicing of mRNA transcripts of the HMG-I/Y gene. They can change the chromatin structure and are auxiliary structural transcription factors (Hill and Reeves, 1997; Hill et al., 1999). HMG-I(Y) binds to two AT-rich regions in the PRD II and PRD IV in DNA minor grooves (Weiss, 2001) and NF-кB and ATF-2/c-Jun bind to these sequences in major grooves. In addition, they specifically interact with the leucine zipper/alkaline region (bZip) of ATF-2. ATF-2 in turn interacts with NF-кB and binds to CBP/p300 and IRFs complex. Ultimately, the IRFs form enhancers with HMG-l(Y), NF-кB, and ATF-2/c-Jun (AP-1) complexes (Du et al., 1993; Figure 3B). Each element must be precisely arranged on the surface of the DNA helix. The interaction among different elements in the enhancer depends on the relative positions of their binding sites and is essential for correct functioning (Zago et al., 2009; Burns and Kerppola, 2012). HMG-I(Y) itself does not activate IFNβ transcription but induces conformational changes in the multi-protein complex. Hence, it is necessary for the transcriptional activity of NF-кB and ATF-2/c-Jun and is also required for transcription to occur (Thanos and Maniatis, 1995).

## 4. Nucleosome remodeling and transcriptional initiation

The nucleosomal chromatin modifications involved in the transcriptional activation of IFNβ fall into two categories: ATP-dependent nucleosomal remodeling and histone acetylation. When IFNβ is activated, the chromatin remodeling complex induces histone transfer and nucleosome sliding. SWI/SNF, an ATP-dependent chromatin remodeling complex, is a multi-subunit assembly composed of eight or more polypeptides, in which BRG1 and BRM1 are the DNA-dependent ATPases. However, this complex lacks sequence specificity in DNA binding, hence it needs to work along with DNA-binding proteins after being recruited to a specific promoter (Agalioti et al., 2000; Gopinathan and Diekwisch, 2022).

IFNβ transcription begins with the correct assembly of enhancers. Next to the enhancer-binding region is nucleosome II, which starts at 5 bp downstream of the TATA box and extends to the transcription initiation site (Goodbourn and Maniatis, 1988; Lomvardas and Thanos, 2001), and thus masks them (Segal et al., 2006). Therefore, IFNβ activation requires the processing of this nucleosome. First, the GCN5 complex is recruited and acetylates the histone amino-terminal tail of nucleosome II (Agalioti et al., 2000; Martinez de Paz and Josefowicz, 2021). Then, the enhancer targets nucleosome II and immediately recruits CBP/PolI holoenzyme complex (Au-Yeung and Horvath, 2018). However, lncRNA BART may hinder this process (Verhoeven et al., 2019). Next, CBP recruits the SWI/SNF complex that remodels nucleosome II. BRG1 binds to the promoter and has a bromodomain that interacts with the N-terminal of acetylated histones, making the binding to nucleosomes more stable. The contact between SWI/SNF-modified histones and DNA leads to distortion and superhelicity of DNA around the histone core without changing the position of the histone core relative to DNA. These changes are sufficient to allow TBP to bind to the nearby TATA box and this completes the assembly of the basic structure. Finally, TBP-induced DNA bending causes the nucleosome to slide 36 bp downstream, thus completely exposing the transcriptional initiation site (Lomvardas and Thanos, 2001). This process is well-correlated with the level of gene induction (Gjidoda et al., 2014). Thus, the assembly process of the IFNβ gene transcription initiation complex is completed and transcription begins (Figure 3C).

## 5. Post-transcriptional regulation

The eukaryotic translation is promoted by the binding of eukaryotic initiation factor 4F (eIF4F) to the 5′ cap structure (m7GpppN, where N is any nucleotide and m is methyl). eIF4F is a three-subunit complex composed of eIF4E, eIF4G, and eIF4A. The eIF4E subunit interacts with the cap structure, eIF4G is a scaffold protein, and eIF4A is a DEAD-box RNA helicase. eIF4F recognizes the hat structure and recruits the 40s ribosomal subunit to the 5′ end of mRNA, thereby completing the 80s ribosomal assembly at the start codon. eIF4F has a specific expression number, which is a translation rate limit point. In addition, the homologous protein 4EHP (eIF4E2) of eIF4E binds to the cap structure but does not interact with eIF4G, hence translation cannot be started. Therefore, 4EHP is an mRNA translation inhibitor (Xu Z. et al., 2022). Moreover, increased IFNβ expression has been found to upregulate miR-34a. Subsequently, miR-34a cooperates with 4EHP to induce translation silencing of IFNβ mRNA through its 3′ non-coding region, which is a negative feedback mechanism to control IFNβ expression. In addition, many miRNAs, such as let-7b, miR-26a, and miR-145, inhibit IFNβ protein (Zhang X. et al., 2021). Xu Z. et al. (2022) found, SARS-CoV-2 encodes the nonstructural protein 2 (NSP2), which specifically binds to the 860–919 amino acid region of the LHR region of Grb10-interacting GYF (glycine, tyrosine, phenylalanine) protein 2 (GIGYF2). This region is a single long alpha helix predicted by AlphaFold 2. Additionally, the N-terminal binding motif of GIGYF2 enhances the binding of 4EHP to the cap structure of IFNβ, leading to the formation of ternary complexes that hinders the translation of IFNβ mRNA. As a result, SARS-CoV-2 can regulate the expression of IFNβ and achieve immune escape.

## 6. Discussion

This review describes the structural elements of the IFNβ promoter, various transcription factors involved in its regulation, the assembly process of IFNβ enhancers, nucleosome remodeling, and post-transcriptional regulation. Generally speaking, the structure and transcription factors of the IFNβ promoter are the key to its regulation. Current research has revealed its proximal as well as distal structure; however, with uncertain positions. The discovery is mainly the enhancer element, but the existence of a suppressor structure is not ruled out. In addition, various hot epigenetic modifications have been identified in the whole regulatory network of IFNβ in recent years, and it is believed that there will be numerous discoveries in the future as well.

Additionally, the signaling pathways and regulatory factors of IFNβ have not yet been completely identified and require more attention. Maintaining the correct expression of IFNβ is of great significance to the life activities of the body, but it is difficult to achieve completely correct regulation. When a link within the INFβ regulatory network goes wrong, it can have a serious impact on the body. For instance, mutations in IFIH1, TREX1, or ADAR1 can cause diseases such as systemic lupus erythematosus (SLE), Aicardi-Goutières syndrome (AGS), and multiple sclerosis (MS). Functional mutations of the USP18 gene can cause TORCH syndrome, while Proteasome-associated autoinflammatory syndrome (PRAAS) and COPA syndrome are also linked to dysregulation of the INFβ pathway. Moreover, many viral escape mechanisms are achieved by destroying IFNβ regulation, for example, immunodeficiency disease caused by viral infection (Collado-Hidalgo et al., 2006). Additionally, accumulating studies have shown that tumorigenesis is closely related to abnormal regulation of IFNβ (Jha et al., 2022). Therefore, maintaining IFNβ expression, in a reasonable range, is of immense significance for experimental research and disease treatments. Hence, many researchers are using the IFNβ regulatory network as the target of agonists or inhibitors in scientific research and the therapeutic target of related diseases (Lin et al., 2011; Malik et al., 2015; Hernández-Díaz et al., 2021).

However, using the IFNβ regulatory network as a therapeutic strategy has limitations due to the complexity of the mechanism involved in regulating its expression. Many mechanisms can participate in this regulating, and targeting IFNβ alone may not be sufficient. Developing a drug that can specifically target the rate-limiting step that affects INFβ expression is challenging, but essential to achieve the desired therapeutic effect. The complexity of the regulatory mechanism can also lead to the development of drug resistance in cells, which can be detrimental to the long-term treatment of interferon-related diseases. For instance, studies have shown that the ADAR1 gene can edit dsRNA to block the recognition of related proteins, inhibiting the interferon signal pathway and causing cells to develop drug resistance. This is particularly disadvantageous for treating tumors and other related diseases. Therefore, in drug development in this area, we should strive to find ways to avoid such resistance (Ishizuka et al., 2019).

## Author contributions

QC and JZL proposed the idea of the review, while JF, QL, JDL, ZC, and LC collected and sorted out the literature. JF wrote the first draft, while QC revised the manuscript. All authors contributed to the article and approved the submitted version.

## Funding

This study was financially supported by the University-Industry Cooperation Project of Fujian Province, China (Grant No. 2021N5003).

## Conflict of interest

The authors declare that the research was conducted in the absence of any commercial or financial relationships that could be construed as a potential conflict of interest.

## Publisher’s note

All claims expressed in this article are solely those of the authors and do not necessarily represent those of their affiliated organizations, or those of the publisher, the editors and the reviewers. Any product that may be evaluated in this article, or claim that may be made by its manufacturer, is not guaranteed or endorsed by the publisher.
